# Proinflammatory cytokines in Egyptian elderly with chronic obstructive pulmonary disease

**DOI:** 10.4103/0970-2113.71956

**Published:** 2010

**Authors:** Moatassem S. Amer, Hoda M.F. Wahba, Samiha S.A. Ashmawi, Randa Reda Mabrouk, Ahmad A.E. Sharaf Eldeen, Sarah A. Hamza

**Affiliations:** *Department of Geriatric, Egypt*; *Department of Pulmonary Medicine, Ain Shams University, Cairo, Egypt*; *Department of Clinical Pathology, Ain Shams University, Cairo, Egypt*; *Department of Cardiology, Ain Shams University, Cairo, Egypt*

**Keywords:** Chronic obstructive pulmonary disease, C-reactive protein, interleukin-1 beta, cytokines, tumor necrosis factor-α

## Abstract

**Background:**

The pulmonary component of chronic obstructive pulmonary disease (COPD) is characterized by airflow limitation that is not fully reversible. The airflow limitation is usually progressive and associated with an abnormal inflammatory response of the lung to noxious particles or gases.

**Hypothesis:**

The levels of the proinflammatory cytokines, interleukin 1 beta (IL-1β), tumor necrosis factor alfa (TNF-α), and C-reactive protein (CRP), in elderly patients suffering from COPD are increased.

**Settings and Design:**

A case control study involving 90 elderly participants from the outpatient clinics of Ain Shams University hospitals.

**Materials and Methods:**

The 90 subjects were subdivided into three equal groups ’ group I (control), group II (patients with COPD), and group III (patients with COPD and cardiovascular complications). Comprehensive clinical assessment, pulmonary functions, and echocardiography were performed. The levels of IL-1β, TNF-α, and CRP were measured in the patients’ serum and compared.

**Statistical analysis:**

SPSS (Statistical Package for Social Science) version 10.

**Results:**

IL1-βand CRP were significantly higher in the third group than the first group (*P* <0.05). There was a similar significant difference between the second and third group as regards IL1-βand CRP (*P* < 0.05). Positive significant correlation between CRP and TNF-α with stage of COPD according to FEV1 (*P* <0.05) were found.

**Conclusions:**

Complicated cases of COPD had higher levels of IL1-β and CRP and the more severe the cases, the higher the levels of CRPand TNF-α.

## INTRODUCTION

The pulmonary component of chronic obstructive pulmonary disease (COPD) is characterized by airflow limitation that is not fully reversible. The airflow limitation is usually progressive and associated with an abnormal inflammatory response of the lung to noxious particles or gases.[1] COPD is basically a benign disease, but the prognosis is so poor thatits mortality rate is similar to that of some malignant diseases.[2] COPD is the fourth leading cause of death in the world, and further increase in its prevalence and mortality can be predicted in the coming decades.[3]

The inflammation involves a multitude of cells, mediators, and inflammatory processes. Activated inflammatory cells release mediators that are capable of damaging lung structures.[4]

Increased levels of TNFα have been reported in sputum and in the circulation of patientswith COPD.[5]

Systemic hypoxemia noted in patients with COPD is associated with acceleration of TNFα productionin alveolar macrophages and peripheral blood mononuclear cells.[6] TNF is an endogenous pyrogen that stimulates the production of other endogenous pyrogens such as IL1β.[7] Levels of CRP are also elevated in patients with COPD.[8]

Chronic hypoxia causes pulmonary hypertension with smooth muscle cell proliferation and matrix deposition in the wall of thepulmonary arterioles. The proinflammatory action of hypoxia is mediatedby the induction of distinct cytokinessuch as IL1β and IL6.[9]

It has recently been reported that TNFα over expression leads to development of severe pulmonaryhypertension and right ventricular hypertrophy.[10]

The aim was to study the proinflammatory cytokines in relation to different groups of COPD in our population to be able to compare it with other populations in whom recent treatments have proven effective.

## MATERIALS AND METHODS

A case control study was conducted and subjects were randomly selected from the outpatient clinics of Ain Shams University hospitals.

The approval of the Geriatric Department, Faculty of Medicine, Ain Shams University was taken. Theconsent to participate in the study for both patients and controls was taken after discussing all the steps of the study and its importance.

A sample of three groups of matched age and sex were allocated. The first group consisted of thirty elderly subjects, 60 years and over with no apparent evidence of disease after full medical history, examination, and selected investigations (control group). The second group consisted of thirty elderly subjects, 60 years and over, suffering from COPD without other comorbid diseases (1^st^ case group). The third group consisted of thirty elderly subjects, 60 years and over, suffering from COPD with cardiovascular involvement without other comorbid diseases (2^nd^ case group). Patients refusing to participate in any of the phases of the study, patients with poor echogenic characteristics, elderly suffering from other comorbid diseases and patients receiving drugs known to affect the proinflammatory cytokines were excluded from the study.

Comprehensive clinical assessment was performed in all the subjects of the three groups. Pulmonary function tests and echocardiography were performed for the members of the three groups to support the clinical data of the patients and correctly categorize them. The levels of IL1β, TNFα and CRP measured were for the three groups in patients’ serum using ACCUCYTE (USA) for measuring Human ILβ, ACCUCYTE (USA) for measuring Human TNFα and DiaMed EuroGen diagnostic kit ELISA (Belgium) for measuring CRP.

The echocardiographic study was performed via transthoracic echocardiography using 2.5-3.5 MH transducer.

The data were analysed using SPSS (Statistical Package for Social Science) version 10, quantitative data were described in the form of mean and standard deviation (SD). Qualitative data were presented in the form of frequency and percentage. Wilcoxon Rank-Sum test (Z value) was used for non-parametric data and Student t test for parametric data. The probability of error (*P* value) was calculated. *P* value was set at 0.05 (*P*>0.05 insignificant, *P*>0.05 significant and *P*>0.01 highly significant). The levels of the three proinflammatory cytokines (IL1β, TNFα, and CRP) and the significance of the difference in cases against control group was hence predicted.

The correlation coefficient (r value) was calculated, and in comparison to the critical value, significant difference between the levels of the proinflammatory cytokines (IL1β, TNFα, and CRP) within each group was identified.

## RESULTS

The study included 28 males and 2 females in each group. There were 19 participants in the age group 60-69 years, 9 participants in the age group 70-79 years and 2 participants in the age group 80-89 years. The control group consisted of 6 smokers, 20 nonsmokers and 4 exsmokers. The COPD group consisted of 24 smokers and 6 exsmokers. The third group consisted of 21 smoker and 9 exsmokers.

There was no significant difference between the levels of IL1-β, TNF-α and CRP in the control group and group II [Table 1].

**Table 1:** Correlation of levels of inflammatory markers between Group I and II

Groups	Group I		Group II		
Inflammatory markers	Mean	SD	Mean	SD	*P* value
IL1β	1.846	2.28	1.185	1.27	0.217
TNFα	12.656	9.06	16.462	19.42	0.404
CRP	10.173	16.32	13.890	25.24	0.234

On the other hand comparing the levels of the inflammatory markers in group I and group III revealed that the level of IL1-β is significantly higher in the third group. There is a nonsignificant difference between the levels of TNF-α in the control group and the third group. CRP is significantly higher in the third group [Table 2].

**Table 2:** Correlation of levels of inflammatory markers between Group I and III

Groups	Group I		Group III		
Inflammatory markers	Mean	SD	Mean	SD	*P* value
IL1β	1.846	2.28	3.228	3.65	0.026
TNFα	12.656	9.06	15.513	14.37	0.375
CRP	10.173	16.32	11.613	14.71	0.033

There is a highly significant difference between the level of IL1-β in group II and group III where its level is higher in group three. There is a nonsignificant difference between the level of TNF-α in group II and group III. CRP is significantly higher in the COPD group [Table 3].

**Table 3:** Correlation of inflammatory markers between Group II and III

Groups	Group II		Group III		
Inflammatory markers	Mean	SD	Mean	SD	*P* value
IL1β	1.185	1.27	3.228	3.65	0.002
TNFα	16.462	19.42	15.513	14.37	0.440
CRP	13.890	25.24	11.613	14.71	0.018

According to Global Initiative for Chronic Obstructive Lung Disease (GOLD), 2006[11] staging of subjects according to the FEV1 was applied for patients in the second and third group [Table 4].

**Table 4:** Percentage of subjects in each stage according to FEV1

	Stage 1 (%)	Stage 2 (%)	Stage 3 (%)	Stage 4 (%)
Group II	43.3	43.3	13.3	0
Group III	0	26.7	23.3	50

Stage 1, FEV1≥80%; Stage 2, FEV1 50–79%; Stage 3, FEV1 30–49%; Stage 4, FEV1 30–49%+respiratory failure/<30%

Positive significant correlations betweeneach CRP and TNFa with stage of COPD according to FEV1 (*P*>0.05) were found.

## DISCUSSION

It is generally accepted that cigarette smoking is the most importantrisk factor for COPD.[2] A difference has always existed in the prevalence of COPD inmales and females, with males having higher rates. The gap betweenmales and females has been narrowing due to the increasedrate of female smoking in the last 20–30years.[12] This explains the marked difference in percentage of males and females in the present study. This is probably because this study includes individuals from a certain socioeconomic sector where the female smokers have not yet increased in number.

The study included 19 subjects in the 60-69 age group, 9 in the 70-79 age group and 2 in the 80-89 age group, in each group studied. In an epidemiologic study of COPD in Canada, the prevalence rate was highest in those persons over age 75.[13] The difference is probably due to the fact that the numbers in the present study may not reflect the true percentages in the community as they were taken from the outpatient clinics of Ain Shams University Hospitals. It may also be due to cultural or other subject related differences between the two countries such as air pollution or duration of smoking.

The mean value of IL-1β in the COPD group was found to be insignificantly less than the control group. According to Joos *et al*,[10] there is an increased release of the proinflammatory cytokine IL-1 from the alveolar macrophages of cigarette smokers and COPD patients. This could explain partially why the control group had a higher mean level than COPD group, as there were smokers in that group, hence elevating IL1β even though the subjects didn’t suffer from COPD. On the other hand, the level of IL1β was higher in the COPD with cardiovascular complication group than the control group. This is in agreement with Finder *et al*,[7] who state that IL1β is a critical sentinel inflammatory cytokine in the pulmonary circulation. There was a highly significant difference in the level of IL1-β in the COPD group and the COPD with cardiovascular complications group where its level is higher in the COPD with cardiovascular complications group. It has also been said that it is apparent that pulmonary vascularsmooth muscle cells are an important target for IL1β.[14]

There was a nonsignificant difference between the mean values of TNFα in the COPD group and the COPD with cardiovascular complications group. When comparing the mean value of TNFα in the control group versus either of the other two groups, a nonsignificant difference was found. It can’t be concluded that the results of this study are against previous studies that state that TNFα expression in patients with COPD may be higher[15] nor are they against the study by Chung[4] that stated that overexpression of TNFα resulted in severe pulmonary hypertension.[16] The control group values in our study could have been raised close to the values of the other groups as smoking in subjects in the control group might have worked as a confounding factor. According to Gander *et al*,[17] plasma levels of tumor necrosis factor (TNF) α is elevated in smokers.[18] It may also be due to the fact that each group contains a wide range of disease progression, and hence classifying the subjects according to disease severity (e.g. FEV1/FVC or RVSP) could have given more accurate results.

It comes to notice that in the three groups the level of CRP varies considerably. Where as in controls it is above the acceptable upper limit in some individuals, in the other two groups is within the normal range in some individuals. This is in agreement with Kelly,[19] who noted that, in healthy controls; there is a wide rangeof CRP values extending well beyond whatwould be considered to be the normal range. He stated that the reason for thisis unclear, but it does suggest that these individuals are notas healthy as described.[20] According to this study there was no significant difference in the level of CRP between the control group and the COPD group, while there was a significant difference in the level of CRP between the control group and the COPD with cardiovascular complication where it was higher in the latter.

Kelly[19] agrees as he describes his results stating that patients with stable COPDhave a range of CRP values that also extend beyond the normalrange. his results are not consistent with previous studies, which suggestthat, in patients with stable COPD, the range of CRP valuesfalls within the normal range. He explains this difference by the possibility of undiagnosed bronchiectasisto have been present. Previous work has shown that 29% of patientspresenting with what appeared to be stable COPD had CT evidenceof at least mild bronchiectasis. This could conceivably explaina wider range of CRP levels. In addition, it is interestingthat after just 5 days of treatment for an acute exacerbationof COPD the CRP had returned to a level below that of the stablecohort in the study. Since standard treatment for an exacerbationis able to achieve this in just a few days, it suggests thatthe stable group may have contained individuals that were infact not so stable.[20] In another study in patients with pulmonary hypertension, serum CRP levels were significantly higher than inthose patients without hypertension.[21]

The group of individuals with COPD with cardiological complication in our study includes a wide range of severities, starting from those clinically just COPD but have echocardiographic changes suggesting cardiologic affection, up to those with COPD with corpulmonale. This explains why the values in this group range from values in the normal range, as they are clinically stable COPD, to values well beyond that range.

The difference in results of this study when compared with other studies could also be attributed to the difference of age groups studied. Several studies have demonstrated that an increased inflammatory activity accompanies ageing. In fact, old individuals show 2–4 fold increased plasma and serum levels of inflammatory mediators such as cytokines and acute phase proteins. There is a well documented basal low grade pro-inflammatory activity in the elderly as compared to young people. It is possible that a wide range of factors contribute to this basal low grade inflammation, including an increased amount of fatty tissue, smoking and subclinical infections. Alternatively, the proinflammatory status observed in older persons might depend on the chronic antigenic stress which bombards the innate immune system thorough out life. Old people have had to cope with a lifelong antigenic burden encompassing several decades of evolutionary but unpredictable antigenic exposure.[22]

According to Gan *et al*,[8] smoking elevates inflammatory markers even when lung function is not impaired. Perhaps, the toxins start the initial inflammatory response and as smoking continues, precipitate a continued low-grade systemic inflammation that worsens lung function and contributes to other complications over time. Further research is needed to elucidate the interaction of these inflammatory markers with the lungs and vascular systems. In addition, as reported, a prospective study is required to define the temporal relationship between the markers, smoking, and reduced lung function.[23] Since smoking is an important factor affecting the proinflammatory cytokines as an independent factor, its effect on the cytokines couldn’t be statistically studied in this study as there were no matched numbers of smokers in each group. To study its independent effect, it is essential to study smokers without any comorbid diseases in comparison to smokers with COPD and COPD patients due to causes other than smoking.

Chemokines have significant redundancy. Inflammatory cytokines do share some intracellular signaling pathways, suggesting that agents that target signaling systems could have broad effects on inflammation.[24] Therefore, the present study tried to find a correlation between the proinflammatory cytokines within each group.

Regarding the COPD group in our study, there is a positive significantcorrelation between CRP and stage of COPD according to FEV1. This is in agreement with the study by Sin and Paul Man,[22] where participants with severe airflow obstruction (defined as FEV _1_ <50% of predicted) were 2.74 times more likely to have highly elevated (CRP >10.0 mg/L) serum CRP levels than those without airflow obstruction after adjustments for a variety of factors, including age, gender, smoking history, body mass index, and comorbidities. Participants with moderate airflow obstruction (defined as FEV _1_ 50%-80% of predicted) were 1.56 times more likely to have highly elevated serum CRP levels.[25] Regarding the group with COPD and cardiovascular complications in the present study, there is also a positive correlation between TNF-α and stage of COPD according to FEV1 in the COPD and cardiovascular complications group.
